# Consistency in knee MRI reporting templates: an ISS survey and common data element analysis

**DOI:** 10.1007/s00256-026-05231-1

**Published:** 2026-04-29

**Authors:** Maximilian Paul, Fabio Dennstädt, Paul Martin Putora, Sebastian Leschka, Tim Steffen Fischer, Reto Sutter, Tobias Johannes Dietrich

**Affiliations:** 1 HOCH, Cantonal Hospital St. Gallen, Radiology and Nuclear Medicine, Rorschacherstrasse 95, 9007 St. Gallen, Switzerland; 2 Faculty of Medicine, University of Zurich, Pestalozzistrasse 3, 8091 Zurich, Switzerland; 3 Department of Radiation Oncology, Inselspital, Bern University Hospital and University of Bern, Freiburgstrasse 18, 3010 Bern, Switzerland; 4 Department of Radiation Oncology, HOCH, Cantonal Hospital St. Gallen, Rorschacherstrasse 95, 9007 St. Gallen, Switzerland; 5 Division of Radiology and Nuclear Medicine, Cantonal Hospital Winterthur, Brauerstrasse 15, 8401 Winterthur, Switzerland; 6 Department of Radiology, Balgrist University Hospital, Forchstrasse 340, 8008 Zurich, Switzerland

**Keywords:** Knee joint, Magnetic resonance imaging, Common data elements, Reporting, Template

## Abstract

**Objective:**

This study aimed to analyze knee MRI reporting templates among radiologists of the International Skeletal Society (ISS) and to determine standardized information in the form of common data elements.

**Materials and methods:**

A total of 510 radiologists, all members of the ISS, were invited to complete a survey and submit knee MRI reporting templates used in clinical practice. Each template was analyzed for structure and common data elements, as defined by the National Institutes of Health. Common data elements were classified as value list, text, or number. Multi-option value list elements were recoded into binary values. The results were aggregated to quantify the prevalence of individual common data elements and groups.

**Results:**

A total of 73 of 510 (14%) invited ISS members completed the survey. Forty-one respondents (56%) reported using templates, whereas 32 (44%) did not. Thirty-nine respondents submitted 62 templates from 22 countries. Templates originated mainly from Europe (38%), North America (31%), and Asia (18%). Among respondents who submitted templates, 49% used highly structured templates, 33% intermediate-structure templates, and 18% low-structure templates. Most intermediate templates were anatomy-based (62%) rather than compartment-based (38%). A total of 507 common data elements were extracted, comprising 75% value list elements, 23% text elements, and 2% number elements.

**Conclusion:**

Knee MRI reporting in this sample of ISS respondents was not standardized, with only a slight majority reporting template use and substantial heterogeneity in template structure. The analysis identified essential and consistently reported items.

**Supplementary Information:**

The online version contains supplementary material available at https://doi.org/10.1007/s00256-026-05231-1.

## Introduction

Standardized reporting in MRI has been shown to improve the consistency of reporting and overall diagnostic quality [1–6]. Various studies have reported the benefits of using reporting templates. However, the implementation of knee MRI reporting templates seems inconsistent and remains a controversial topic [7]. Common data elements (CDEs) are defined by the National Institutes of Health CDE Repository as standardized reusable questions with a predefined set of answers that can be used across systems and studies [8]. They were introduced to facilitate consistent and systematic data collection across different sites within a clinical trial but are increasingly being applied to collection of real-world data. CDEs can help convert prose into computable data units. In knee MRI reporting, examples include value list items such as ACL integrity (e.g., intact, partial tear, complete tear), text items such as a free-text description of meniscal status, and number items such as the TT-TG distance or Insall-Salvati ratio. Illustrative examples of CDEs and their classification are provided in Table 1. Reporting and research in radiology may benefit from the use of standardized CDEs that can be shared across systems and studies [8].

**Table 1:** Illustrative examples of common data elements in knee MRI reporting

Example CDE	CDE type	Example values/entry format	Example purpose in knee MRI reporting
ACL Integrity	Value list	intact/partial tear/complete tear	Structured assessment of anterior cruciate ligament status
Joint Effusion	Value list	none/small/moderate/large	Standardized grading of joint fluid
Menisci Status	Text	free-text entry	Narrative description when findings cannot be reduced to predefined categories
TT-TG Distance	Number	numeric value in mm	Quantitative assessment in patellofemoral instability
Insall-Salvati Ratio	Number	numeric ratio	Quantitative patellar height assessment

Representative examples are shown for the three data element types used in this study: value list, text, and number

To our knowledge, data on current knee MRI reporting template practices among radiologists of the International Skeletal Society (ISS) are limited. In particular, reporting template use and content have not yet been examined through a CDE-based analysis in this setting.

Prior studies on structured reporting in knee MRI and musculoskeletal radiology suggest variability in reporting structure and terminology across radiologists, while also indicating that a shared core of essential reporting elements for the principal structures and disorders of the knee can be expected [1, 2, 5, 9]. In this manuscript, the term “templates” is used for standardized knee MRI reporting formats, and “respondents” is used for individuals who completed the survey.

The primary aim of the present study was to analyze knee MRI reporting templates among radiologists of the ISS and to identify the most consistently used CDEs. Secondary endpoints included the exploration of the distribution of CDE data types, such as the prevalence of templates for special dedicated topics and the use of standardized scores.

## Materials and methods

### Survey

**Fig. 1:**
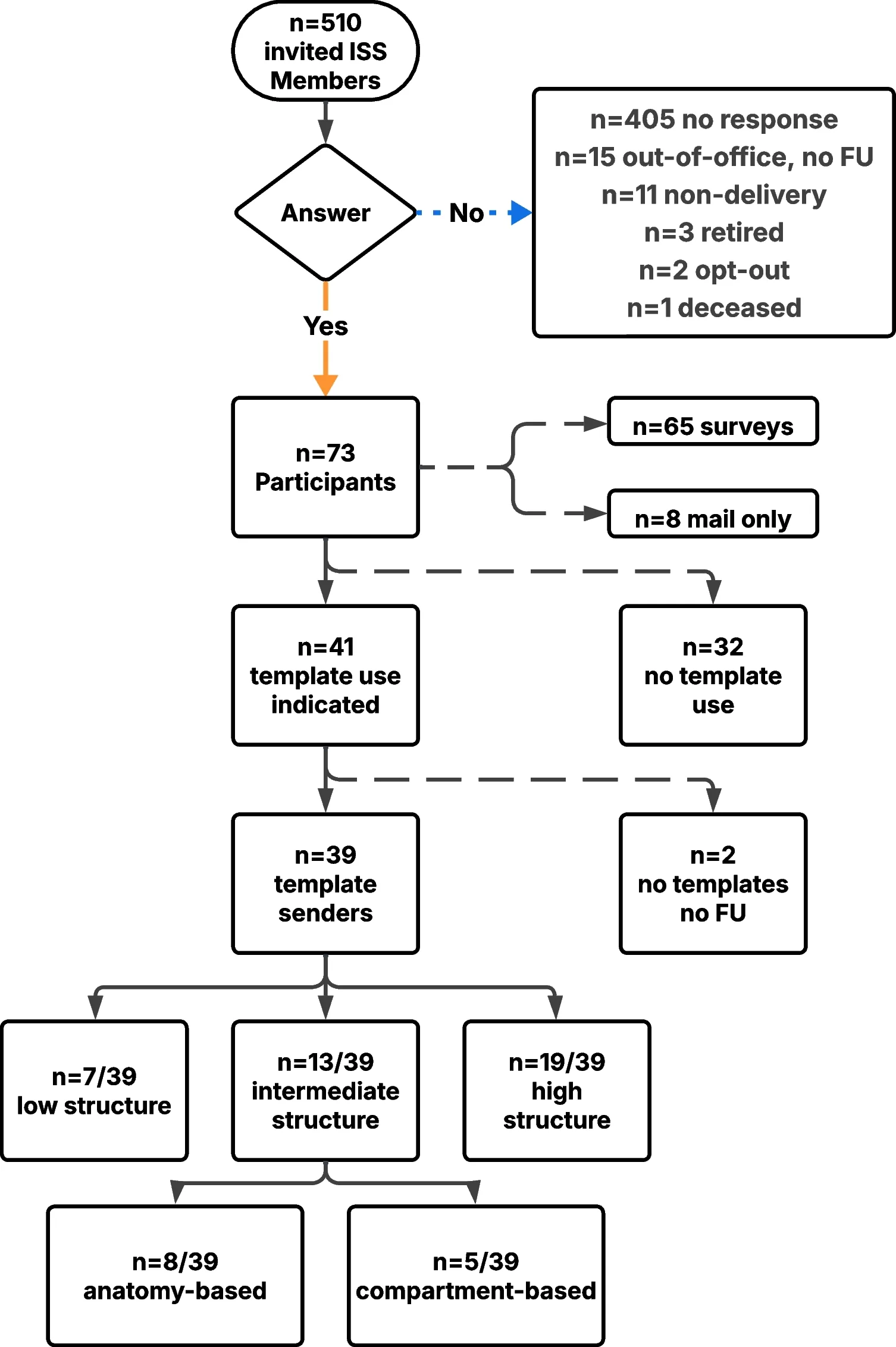
Flow chart of study participation. Response behavior of respondents and distribution of template types. FU = follow-up

Between February and May 2025, a survey of 510 ISS members was conducted using an online survey tool (Jotform, San Francisco, California, U.S.A.). Musculoskeletal radiologists, all members of the ISS, were invited to participate via email through the ISS member directory, accessed in February 2025 [10]. Recruitment was based on the directory and was not randomized. The radiologists were asked to provide templates used at their institution, either via the survey tool or via email (Fig. 1).

The full survey questionnaire is provided in Supplementary Material 1. It was created by the two radiologist study coordinators as a study-specific, non-standardized instrument and was not formally validated. It asked about department-level use and recommendation of knee MRI templates and related reporting practices. All survey items were mandatory, with multiple selections permitted for selected questions. Respondents could complete the survey even if no template was available. A survey response was defined as a completed questionnaire. Department-wide adoption was not independently verified.

To reduce duplicate submissions, when multiple ISS members were affiliated with the same institution, one member was contacted first and additional members were contacted only if no response was received. No reminder was sent to nonresponders. Follow-up contact was limited to respondents who reported template use but had not submitted a template.

### Templates

All received templates were included to achieve a broad overview of the respondents’ reporting practices, and no specific instructions regarding the template form, language, or structure were provided. Non-English templates were translated using DeepL Translator (Cologne, Germany) and returned to the respondents for language verification. The proposed changes were considered in the evaluation process and translations from German were reviewed by native speakers (two investigators). Reporting templates did not undergo further editing.

The templates were classified as either general knee MRI reporting templates or dedicated templates, which were customized for focused reporting on specific disorders.

The classification framework of the reporting structure was adapted from Chundru et al. [1] with minor wording refinements. Instead of classifying templates as free-text, structured with headers, and structured-itemized, they were referred to as ‘low structure’, ‘intermediate structure’, and ‘high structure’. Templates were classified as ‘low structure ‘ if they were mainly free-text based, ‘intermediate structure ‘ if they used structured headers, and ‘high structure ‘ if they were structured and itemized. Intermediate-structure templates were further subcategorized according to their predominant organizational logic as anatomy-based or compartment-based. The operational definitions used in this study and example organization patterns for these categories are provided in Table 2.

**Table 2:** Operational definitions and example organization patterns for reporting structure categories used in this study

Category	Operational definition used in this study	Organization pattern
Low structure	Mainly free-text narrative without consistent internal heading structure	Continuous prose report
Intermediate structure, anatomy-based	Structured by anatomical structures or tissue groups, with grouped narrative under headings	Menisci/Cruciate ligaments/Collateral ligaments/Cartilage/Tendons
Intermediate structure, compartment-based	Structured by joint compartments or regions, with grouped narrative under headings	Medial compartment/Lateral compartment/Intercondylar compartment/Patellofemoral or Anterior compartment
High structure	Highly itemized template with predefined structure-specific fields or entries	ACL: intact/signal/course; Meniscus: shape/tear/extrusion; Cartilage: grade/location

If multiple templates were submitted by one respondent, they were merged into one comprehensive template to avoid the overrepresentation of a few centers in the quantitative analysis of CDEs.

### Common data elements

The templates were investigated for candidate CDEs. CDE extraction required repeated interpretive decisions because of variation in language, structure, semantic phrasing, and degree of granularity across the submitted templates. These decisions included whether a reporting item qualified as a distinct CDE, whether semantically overlapping items should be merged, and whether a given item was more appropriately classified as a value list or text CDE. To maintain internal consistency of these operational decisions across the full dataset, the extraction of CDEs from each template was performed by a single reviewer. A second and third reviewer subsequently verified all extracted items, and any discrepancies in interpretation were resolved by consensus agreement. Because independent duplicate extractions were not performed, formal inter-rater reliability statistics, such as Cohen’s kappa, were not calculated. Additional elements included in the submitted templates, such as items of the document header and footer (e.g., examination date or MRI sequences used), were excluded from further analysis as they did not constitute an essential component of the reporting template.

**Fig. 2:**
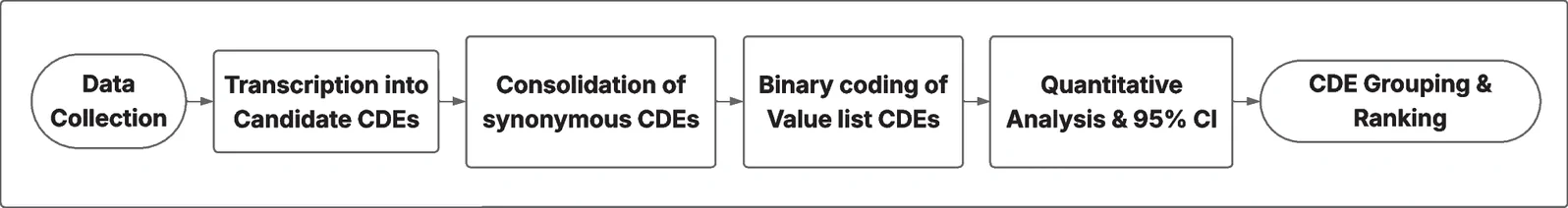
Flow chart of common data element extraction. Step-by-step process of investigation, quantitative analysis, and ranking of common data elements (CDE)

The methodological approach of document analysis to derive CDEs (Fig. 2) was based on previous work by Dennstädt et al. [11]. Any distinct reusable reporting item was considered a candidate CDE, and the original wording and permissible values were retained. Semantically identical or widely overlapping CDEs (e.g., “joint_effusion_size” and “effusion_grade”) were merged under one common CDE by one reviewer, and the extracted items were verified by a second reviewer. The extracted items were grouped into three CDE types, as defined by the National Institutes of Health: value list, text, and number [12].

Value list CDEs were defined as parameters with a predefined set of permissible nominal or ordinal values (e.g., “joint effusion size” with the values “none”, “small”, “moderate” and “large”). Many of the value list CDEs contained more than two possible values. For these candidate CDEs, each value was binarized using the variables “TRUE” or “FALSE” to reduce the specific CDE to answerable binary questions, such as “joint_effusion_none”, “joint_effusion_small”, “joint_effusion_moderate” and “joint_effusion_large”. To quantify the occurrences of a specific CDE among all templates, the value list CDEs were recoded into binary (yes/no) items with a value of 1 for an occurrence in a single template and 0 for a non-occurrence. This was done for all extracted CDEs and then summarized to count the overall presence of a certain CDE among all submitted templates. Text CDEs captured narrative input that could not be reduced to predefined categories or numeric values, and number CDEs comprised numeric values, radiologic ratios (e.g., the Insall-Salvati ratio), and angular measurements (e.g., the trochlear sulcus angle).

### Statistical analysis

A CDE was counted only when it occurred in templates from two or more respondents to rule out the individual preferences of a single respondent. CDEs were then assigned to hierarchical semantic groups (main and subcategories) by the two radiologist study coordinators.

Main categories were defined as the essential anatomical structures of the knee. Subcategories were defined as more specific anatomical substructures or reportable aspects within a main category. Diagnosis- or finding-oriented items (e.g., fractures, osteoarthritis, joint fluids) were presented as a separate block, distinct from anatomical main categories and anatomical substructures/aspects. The complete list of main categories and subcategories, along with a ranking of CDE groups with their respective percentages is provided in Table 3. Higher percentages indicate more frequently included components of knee MRI reporting templates.

**Table 3:** Frequency ranking of common data element (CDE) groups across submitted knee MRI templates (n = 39), organized into anatomical main categories (A), anatomical substructures/aspects (B), and a separate block for findings and diagnoses (C). n = number of respondents whose submitted template(s) contained the specific group. 95% confidence intervals (CI) were calculated using the Wilson score method

CDE group	n	Frequency	Lower 95% CI	Upper 95% CI
**A. Anatomical domains (main categories)**				
Bones	39	100.0%	0.91	1.00
Menisci (NOS)	37	94.9%	0.83	0.99
Joint & Popliteal Space	35	89.7%	0.76	0.96
Cartilage (NOS)	34	87.2%	0.73	0.94
Cruciate Ligaments	34	87.2%	0.73	0.94
Patellofemoral Joint	34	87.2%	0.73	0.94
Collateral Ligaments	32	82.1%	0.67	0.91
Muscles & Soft Tissues	25	64.1%	0.48	0.77
Periarticular	16	41.0%	0.27	0.57
Bursae	13	33.3%	0.21	0.49
Neurovascular	7	17.9%	0.09	0.33
**B. Anatomical substructures (subcategories)**				
Anterior Cruciate Ligament	33	84.6%	0.70	0.93
Extensor Apparatus	32	82.1%	0.67	0.91
Posterior Cruciate Ligament	32	82.1%	0.67	0.91
Bone Morphology and Marrow	31	79.5%	0.64	0.89
Lateral Collateral Ligament	31	79.5%	0.64	0.89
Lateral Meniscus	31	79.5%	0.64	0.89
Medial Collateral Ligament	31	79.5%	0.64	0.89
Medial Meniscus	31	79.5%	0.64	0.89
Joint Compartments and Articulation	24	61.5%	0.46	0.75
Patellofemoral (anterior) Compartment Cartilage	22	56.4%	0.41	0.71
Medial Compartment Cartilage	21	53.8%	0.39	0.68
Lateral Compartment Cartilage	19	48.7%	0.34	0.64
Anterior Soft Tissues	17	43.6%	0.29	0.59
Posterolateral Complex	16	41.0%	0.27	0.57
Patellofemoral Morphology & Alignment	13	33.3%	0.21	0.49
Anterolateral Complex	11	28.2%	0.17	0.44
Nerves	7	17.9%	0.09	0.33
Meniscal Stabilizers	4	10.3%	0.04	0.24
Meniscocapsular Structures	4	10.3%	0.04	0.24
Posteromedial Complex	4	10.0%	0.04	0.24
Vascular	3	7.7%	0.03	0.20
**C. Findings and diagnoses**				
Joint Fluids	33	84.6%	0.70	0.93
Popliteal Cyst	17	43.6%	0.29	0.59
Popliteal Space Other	14	35.9%	0.23	0.52
Fractures	13	33.3%	0.21	0.49
Patellar Stabilizers & Instability	13	33.3%	0.21	0.49
Synovia	12	30.8%	0.19	0.46
Osteoarthritis	9	23.1%	0.13	0.38
Other	9	23.1%	0.13	0.38

For each CDE, two-sided 95% confidence intervals were calculated using the Wilson score method with z = 1.96, and no continuity correction was applied. The proportion was defined as $\widehat{p} =\frac{\mathrm{x}}{\mathrm{n}}$ where $\widehat{p}$ is the sample proportion, *x* is the number of respondents whose submitted template(s) contained the CDE and *n* the number of evaluable respondents (n = 39). Statistical analyses were performed using R (R Foundation for Statistical Computing, Vienna, Austria). Data handling and table preparation were conducted using Microsoft Excel (Microsoft Corporation, USA), and flowchart visualizations were created using Lucid (Lucid Software Inc., South Jordan, UT, USA). During manuscript preparation, a large language model was used solely for language editing and phrasing refinement.

## Results

### Survey

A total of 73 of 510 invited ISS members (14%) completed the survey. Forty-one respondents (56%) reported using at least one template, while 32 (44%) reported no template use. Two respondents reported using templates but did not submit any files despite follow-up. Therefore, 39 respondents who submitted templates were included in the analysis.

In total, 62 templates were obtained from 39 (53%) respondents, averaging 1.6 templates (median = 1; IQR = 0) per respondent. Templates were received from 22 different countries (Argentina, Belgium, Brazil, Canada, Colombia, Egypt, France, Germany, Greece, India, Italy, Japan, Netherlands, New Zealand, Qatar, Singapore, Spain, Sweden, Switzerland, Turkey, UK, and USA). One respondent provided independent data for institutions from two countries and was therefore counted as two separate entities. Of 510 invitation emails, 405 (79%) received no response. Eleven (2%) emails could not be delivered. Fifteen emails (3%) received automatic out-of-office replies with no subsequent follow-up. Three recipients (< 1%) subsequently indicated that they were already retired, two recipients opted out of further contact, one recipient had passed away.

### Geographical distribution of template use

The USA was represented the most, with 10 of 12 respondents (83%) using a template. The United Kingdom had nine respondents; one respondent (11%) reported using a template, while seven respondents reported no use, and one respondent indicated template use but did not submit a template. From Canada, two out of six respondents (33%) used a template. From Germany, four out of six (67%) respondents used a template. France had five respondents, one used a template (20%), three did not, and one indicated template use but did not supply the template. In Switzerland, two of five respondents (40%) used a template. In Greece, two out of four respondents (50%) used a template. Among the two respondents from Finland, none reported using a template. Overall, 50% of respondents from Italy, Japan, and the Netherlands (1/2 from each country) and 100% of respondents from India and Turkey (2/2 from each country) reported using templates. Argentina, Belgium, Brazil, Colombia, Egypt, New Zealand, Qatar, Singapore, Spain, and Sweden each had one respondent who used a template (100% each). Australia, Chile, Hong Kong, and Israel each had one respondent, and none used a template (0%).

This resulted in 15 respondents from Europe (38%), 12 from North America (31%), seven from Asia (18%), three from South America (8%), one from Africa (3%), and one from Oceania (2%). No duplicate templates were received. Among respondents who submitted templates, 30/39 (77%) relied on a single template for all knee MRI examinations. Rarely, two closely related versions were used, typically to distinguish laterality or level of detail.

### Templates

A total of 19 templates with special dedicated topics were obtained. The topic patellar instability was found in templates of four respondents. Two respondents used a dedicated template for anterior cruciate ligament tears. A dedicated template for degenerative arthropathy was found for two other respondents, with slight variations in the focused anatomical structures. Other dedicated topics included anterior cruciate ligament grafts, bone-tendon-bone grafts, meniscal tears, degenerative meniscectomy, postoperative meniscus, post-traumatic joint disorders, and juvenile idiopathic arthritis. These topics were always limited to one respondent and were not represented by other respondents.

Templates translated using DeepL were returned to the respective respondents for verification that the translated content remained semantically accurate. Only minor corrections were received during this verification step and were considered in the analysis. Twenty-three respondents submitted templates that included document header and/or footer elements, whereas 16 submitted only the core reporting template without additional elements. Only one respondent reported using the template recommended by their national radiological society. This respondent was also the author of that exact template. Every other respondent submitted their individual templates. Thus, no shared “consensus” template among several ISS members, such as from RadReports.org by the RSNA [13], was used.

Of the 62 templates received, 13 (21%) met the modified Chundru [1] criteria for low structure, 23 (37%) had an intermediate structure, and 26 (42%) were highly structured. Among the intermediate templates, 18 (78%) were anatomy-based and five (22%) were compartment-based. Among respondents who submitted templates (n = 39), nearly half used highly structured templates (19/39, 49%). Intermediate-structure templates were used by 13/39 respondents (33%), most of which were anatomy-based (8/13) rather than compartment-based (5/13). Low-structure templates were least common (7/39, 18%). Respondents who used multiple templates consistently used the same overall structure category (low, intermediate, or high). Within the intermediate-structure category, the same predominant organizational format was maintained.

### Common data elements

**Fig. 3:**
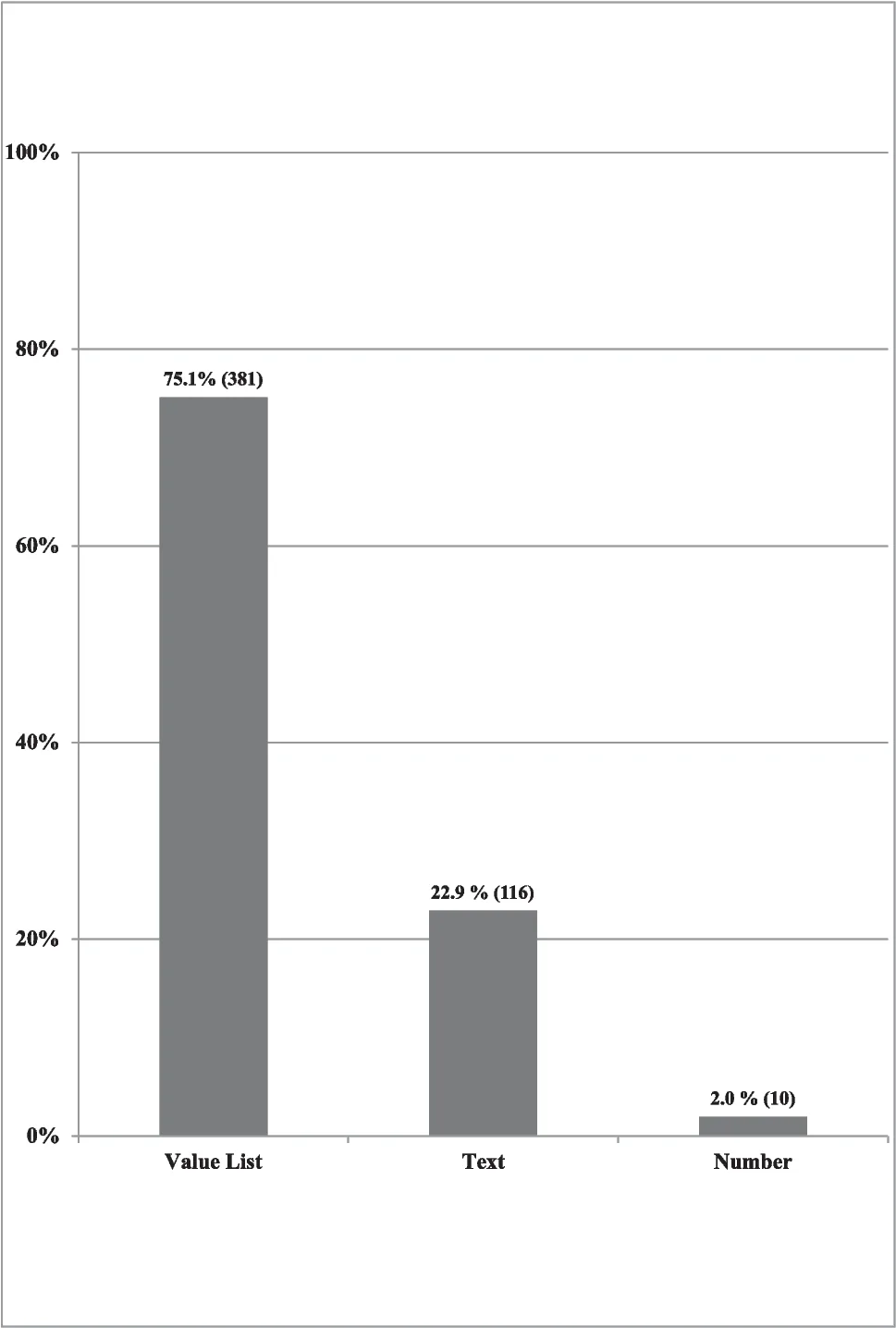
Absolute and relative distribution of the extracted common data element types among all submitted templates

After aggregating identical duplicates and excluding the contents of the document header and footer, 507 individual CDEs were extracted from all templates. Overall, 381 value list CDEs (75%), 116 text CDEs (23%), and 10 number CDEs (2%) were identified (Fig. 3). Number CDEs comprised specific radiological scores or measurements, such as the patellar angle, Insall-Salvati ratio, Caton-Deschamps index, trochlear sulcus angle, lateral trochlear inclination, trochlear facet asymmetry, trochlear depth, TT-TG distance, TT-PCL distance, and Baker’s cyst size. CDEs were often represented at different levels of granularity. Consequently, the same anatomical structure could appear as a value list CDE in one template and as a text CDE in another, depending on the context (e.g., bone marrow edema). Umbrella entries without further specification (e.g., ‘menisci’) were classified as text CDEs and labeled ‘not otherwise specified’ (NOS).

Table 3 summarizes the frequency of CDE groups organized into anatomical main categories, anatomical substructures/aspects, and a separate block of findings/diagnoses (sections A–C). The 50 most common single CDEs are summarized in Table 4. Across anatomical main categories, bones were included in 100% of templates, menisci (NOS) in 95%, and the joint & popliteal space domain in 90% (Table 3 Section A). Cartilage (NOS), cruciate ligaments, and the patellofemoral joint were each present in 87% of templates, followed by collateral ligaments in 82% and muscles & soft tissues in 64%. Periarticular structures were included in 41%, bursae in 33%, and neurovascular structures in 18% of templates.

**Table 4:** Frequency ranking of the 50 most common individual common data elements (CDEs)

Common Data Element	Type	Templates with CDE (n = 39)	Frequency [%]	Lower 95% CI	Upper 95% CI
Joint_Effusion_Presence	Value List	19	48.7	33.9	63.8
ACL_Intact	Value List	18	46.2	31.6	61.4
PCL_Intact	Value List	17	43.6	29.3	59.0
MCL_Intact	Value List	17	43.6	29.3	59.0
LCL_Intact	Value List	17	43.6	29.3	59.0
Menisci_Status	Text	13	33.3	20.6	49.0
Medial_Meniscus_Tear	Value List	13	33.3	20.6	49.0
Patellar_Tendon_Intact	Value List	13	33.3	20.6	49.0
Medial_Meniscus_Shape_Normal	Value List	12	30.8	18.6	46.4
Medial_Meniscus_Signal_Normal	Value List	12	30.8	18.6	46.4
Lateral_Meniscus_Shape_Normal	Value List	12	30.8	18.6	46.4
Lateral_Meniscus_Tear	Value List	12	30.8	18.6	46.4
Quadriceps_Tendon_Intact	Value List	12	30.8	18.6	46.4
Joint_Effusion_Status	Text	12	30.8	18.6	46.4
Bones_Status	Text	11	28.2	16.5	43.8
Lateral_Meniscus_Signal_Normal	Value List	11	28.2	16.5	43.8
Ligaments_Status	Text	11	28.2	16.5	43.8
Extensor_Apparatus	Text	11	28.2	16.5	43.8
Bakers_Cyst_Presence	Value List	11	28.2	16.5	43.8
Fracture_Presence	Value List	10	25.6	14.6	41.1
MCL_Status	Text	10	25.6	14.6	41.1
Medial_Compartment_Cartilage_Normal	Value List	9	23.1	12.6	38.3
Lateral_Compartment_Cartilage_Normal	Value List	9	23.1	12.6	38.3
ACL_Status	Text	9	23.1	12.6	38.3
LCL_Status	Text	9	23.1	12.6	38.3
Extensor_Apparatus_Intact	Value List	9	23.1	12.6	38.3
Intra_Articular_Free_Bodies_Presence	Value List	9	23.1	12.6	38.3
Bone_Marrow_Edema_Presence	Value List	8	20.5	10.8	35.5
Lateral_Compartment_Status	Text	8	20.5	10.8	35.5
Medial_Meniscus_Normal	Value List	8	20.5	10.8	35.5
PCL_Status	Text	8	20.5	10.8	35.5
ITB_Normal	Value List	8	20.5	10.8	35.5
Hoffa_Body_Normal	Value List	8	20.5	10.8	35.5
Medial_Compartment_Status	Text	7	17.9	9.0	32.7
Cartilage	Text	7	17.9	9.0	32.7
Articular_Cartilage_Normal	Value List	7	17.9	9.0	32.7
Lateral_Meniscus_Normal	Value List	7	17.9	9.0	32.7
ACL_Signal_Normal	Value List	7	17.9	9.0	32.7
PCL_Signal_Normal	Value List	7	17.9	9.0	32.7
Other_Findings	Text	7	17.9	9.0	32.7
Retropatellar_Cartilage_Normal	Value List	6	15.4	7.2	29.7
Lateral_Meniscus_Status	Text	6	15.4	7.2	29.7
ACL_Course_Normal	Value List	6	15.4	7.2	29.7
PCL_Course_Normal	Value List	6	15.4	7.2	29.7
PCL_Thickness_Normal	Value List	6	15.4	7.2	29.7
Patellar_Retinaculae_Intact	Value List	6	15.4	7.2	29.7
Muscles_Normal	Value List	6	15.4	7.2	29.7
Synovitis_Presence	Value List	6	15.4	7.2	29.7
Bone_Marrow_Edema_Contusion	Value List	5	12.8	5.6	26.7
Bone_Lesion_Presence	Value List	5	12.8	5.6	26.7

Frequencies are based on 39 evaluable respondents; n indicates the number of respondents whose submitted templates contained the specific CDE. Ninety-five percent confidence intervals (95% CI) were calculated using the Wilson score method. The complete CDE list is provided in Supplementary Material 2

Among anatomical substructures/aspects, the anterior cruciate ligament was included in 85% of templates, while the posterior cruciate ligament and extensor apparatus were each included in 82% (Table 3 Section B). Bone morphology and marrow, medial and lateral menisci, and medial and lateral collateral ligaments were each present in 79% of templates. Joint compartments and articulation were addressed in 62%. Cartilage was most frequently specified for the patellofemoral (anterior) compartment (56%), medial compartment (54%), and lateral compartment (49%). Anterior soft tissues were included in 44% of templates. Periarticular complexes were less frequent, with the posterolateral complex present in 41%, the anterolateral complex in 28%, and the posteromedial complex in 10% of templates. Meniscal stabilizers and meniscocapsular structures were each present in 10%. Neurovascular substructures were rarely reported, with nerves present in 18% and vascular structures in 8% of templates.

Within the findings/diagnoses block, joint fluids were reported in 85% of templates, popliteal cysts in 44%, other popliteal space findings in 36%, and synovia in 31% (Table 3 Section C). Fractures and patellar stabilizers & instability were each reported in 33% of templates, while osteoarthritis and other were each present in 23%.

## Discussion

The most important finding of the present study is that a total of 41 respondents (56%) reported using templates, whereas 32 (44%) did not. Nearly half of the respondents who submitted templates (49%) used highly structured templates, one-third (33%) used intermediate-structure templates, and 18% used low-structure templates. Of the 507 extracted CDEs, 75% were value list CDEs, followed by 23% text CDEs and 2% number CDEs.

The present data on knee MRI reporting templates among this sample of ISS respondents demonstrate substantial heterogeneity in both terminology and template structure, yielding numerous minor semantic variants. Despite harmonization, variability persists because of the inherently diverse radiological and anatomical terminology, which is also reflected in the broad 95% Wilson intervals of the CDEs. Coarser aggregation could have increased the apparent agreement but at the cost of potentially obscuring clinically meaningful distinctions. CDEs related to the main anatomical structures of the knee were most frequently included within the templates, indicating shared essential components of knee MRI reporting templates among respondents.

No clear preference for one type of template structure was identified. Specialized dedicated templates were not used broadly. Templates exclusively for patellar instability were reported by a minority of respondents. Respondents varied in what they considered a “template.” Some respondents submitted templates of intermediate or high structure, while others submitted mainly free-text-based templates of low structure that could be considered standardized prose.

Harris et al. 2023 [14] demonstrated potential negative aspects of template-based reporting. Significantly more time was spent viewing the reporting monitor (67.13 s to 112.28 s (attendings) and from 57.38 s to 109.99 s (residents); (*F* = 12.10, *p* = 0.0017), while less time was spent on the diagnostic monitor (236.79 s to 163.40 s (attendings) and 223.55 s to 182.48 s (residents); (*F* = 3.77, *p* = 0.0623). Chundru et al. showed that templates of intermediate structure improve understandability and clarity. In 83% of the cases, clinicians preferred structured templates with headers over free-text, while free-text was considered the most disjointed in 53% of the cases [1]. Rosskopf et al. concluded that any form of structuring is superior to unstructured free-text dictation. While no clear winner of their interventions was found, any of them improved the outcome. For example, clinically relevant discrepancies in reports were reduced from 3.0% to 1.4% [2].

These findings are further supported by the 2023 update of the European Society of Radiology, which recommends viewing structured reporting on a gradient from standardized prose to fully machine-readable reports. As most clinicians prefer structured reports, modular templates to support harmonization without compromising workflow efficiency are recommended, despite only half of the radiologists preferring them [5].

Harmonization of terminology for musculoskeletal radiology has already been suggested by members of the International Skeletal Society as an important step toward authoritative and standardized terminology [15]. However, a glossary of terms for musculoskeletal radiology does not allow the definition of distinct reporting items with clearly determined permissible values (CDEs).

Combining the glossary with CDEs may further improve reporting consistency and comparability for research and computational analyses while reducing idiosyncratic terminology. The Breast Imaging-Reporting and Data System (BI-RADS) showed moderate to good inter-observer agreements, with κ = 0.61–0.69 for core ultrasound descriptors and κ = 0.48–0.50 for key mammographic descriptors [16]. More standardization of radiology reporting may help achieve results like those seen with the measurable success of the implementation of BI-RADS and similar initiatives in imaging.

Systematized Nomenclature of Medicine Clinical Terms (SNOMED CT) and RadLex were not applied to the acquired data because they were outside the scope of this study. However, a subsequent project could utilize the acquired dataset for vocabulary ID mapping, building upon this work. The created list of CDEs could be considered for submission to the RadElement database by the Radiological Society of North America and the American College of Radiology, in alignment with the National Institutes of Health CDE standards. The resulting data could aid in improving research and facilitate automated data extraction if the templates become machine-readable. Further integration of CDEs into knee MRI reporting may help improve reporting consistency, analogous to the quality gains observed after implementation of BI-RADS in breast imaging [16].

The present CDE dataset may serve as a basis for future consensus statements on structured knee MRI reporting in local, regional, multilingual, or international settings [17]. The Delphi technique may be useful for addressing this issue. An iterative Delphi process could be used to evaluate which CDEs should belong to a core set and which may remain optional [17].

### Limitations

This study has some limitations that need to be considered. The modest sample size, the survey turnout of 14%, and the availability of templates from only 39 respondents allow only limited conclusions regarding the overall reporting habits of radiologists and limit the generalizability of the findings. Practice setting (e.g., academic vs. private practice), years of experience, and subspecialty focus were not collected in the survey. Therefore, trends in template use versus non-use by respondent type and the representativeness of the respondent sample could not be fully assessed. The survey was based on a study-specific, non-standardized questionnaire for which no formal validation or pilot testing was performed. Although the survey asked about department-level use and recommendation of templates, department-wide adoption of the submitted templates could not be confirmed for all submissions. Respondents were exclusively ISS radiologists, which may limit generalizability beyond ISS members. Considering the predominantly Western geographic distribution of ISS members, the data are inevitably biased toward Europe and North America. India and the UK are sparsely represented among template submitters, despite having a substantial number of ISS members. Selection bias is possible if radiologists who use templates, or view them favorably, were more likely to respond than those who do not.

The present study evaluated and compared knee MRI reporting templates among various radiologists; however, it did not qualify as a decision‑making analysis because it lacked a clearly defined goal‑oriented framework, explicit identification of alternative options, predefined criteria, trade‑off assessment and weighting, and iterative refinement. Therefore, it was not possible to develop context-specific decision trees based on these data [18].

CDE extraction has subjective elements, and there are many cases in which two or more options may be equally valid at the same time. This is especially true for classifying CDEs into specific types (value list vs. text), merging semantically similar CDEs, and accumulating CDEs into specific groups. In addition, CDE extraction was performed by a single reviewer and subsequently verified by two additional reviewers. Although this approach was chosen to maintain internal consistency of extraction decisions across a highly heterogeneous dataset, it does not eliminate subjective bias. Because no independent duplicate extraction was performed, formal inter-rater reliability was not assessed.

The document footers and headers were excluded from the analysis, which may have resulted in a loss of information, as they may contain information that could be coded into CDEs, e.g., MRI sequences used or the examination date.

In conclusion, the present findings indicate that knee MRI reporting is not highly standardized in this sample of ISS respondents. A slight majority of respondents reported using templates, and substantial heterogeneity in template structure was observed. The CDE analysis identified essential and consistently reported items.

## Supplementary Information

Below is the link to the electronic supplementary material.

Supplementary file1 (PDF 178 KB)

Supplementary file2 (XLSX 27 KB)

## Data Availability

The full dataset is available upon reasonable request.

## Abbreviations

CDE: Common data element
ISS: International Skeletal Society
MRI: Magnetic resonance imaging
NIH: National Institutes of Health
NOS: Not otherwise specified
