# Supplementary material for: Consistency in knee MRI reporting templates: an ISS survey and common data element analysis
Source: Skeletal Radiol. 2026 Apr 29;55(8):2109–21. doi: 10.1007/s00256-026-05231-1 (PMC13649871; doi:10.1007/s00256-026-05231-1)
Supplement: Supplementary file 1 [file 256_2026_5231_MOESM1_ESM.pdf]

Current Practice of Knee MRI Reporting in Radiology

Dear Colleague,

As part of a scientific study we are conducting a survey which aims to gather information on the current practice of knee MRI reporting in various hospitals worldwide.

The goal is to analyze differences and similarities in the use of Common Data Elements (CDEs) as well as in report structuring.

The survey will take approximately **3-5 minutes to complete**.

Your responses will be used exclusively for scientific purposes. If you have standardized report templates, we would appreciate it if you could share them with us either by email or at the end of this form.

The **deadline** for this task is the **15th of April 2025**.

Thank you in advance for your commitment!

Name *

First Name

Last Name

Email *

example@example.com

Name of your department and institution *

Does your hospital use a standardized report template for knee MRIs? *

- ◯ Yes
- ◯ No
- ◯ Other

If yes, are there different templates for specific indications? (Multiple selections possible) *

- ☐ One template for all Knee MRI
- ☐ Orthopaedics & Traumatology
- ☐ Neoplasm
- ☐ Chronic inflammatory diseases
- ☐ No reporting template
- ☐ Other

Are there cases where freestyle text is used instead of a structured template? (Multiple selections possible) *

- ☐ No, we always use structured templates
- ☐ Orthopaedics & Traumatology
- ☐ Neoplasm
- ☐ Chronic inflammatory diseases
- ☐ Other

Is artificial intelligence (AI), including large language models (LLMs), already being used in the knee MRI reporting process at your hospital? *

- ◯ Yes, for image analysis
- ◯ Yes, for report support (e.g. automated structured text generation from freetext)
- ◯ Yes, for image analysis and report support
- ◯ No, but it is planned
- ◯ No, and it is not planned
- ◯ Other

Do final reports for referring physicians or patients include additional visual elements? (Multiple selections possible) *

- ☐ No
- ☐ Example images from the MRI
- ☐ Link to a summary video
- ☐ Other

Please submit us your knee reporting templates below, if available (alternatively by email). *

Do you have any further comments regarding our study or structured knee MRI reporting? (Free text field)

Thank you very much for your time and commitment!
